# Comparative Evaluation of ChatGPT and Gemini in Approximating Clinically Confirmed Diagnoses From Structured Numerical Pure-Tone Audiogram Data

**DOI:** 10.7759/cureus.111153

**Published:** 2026-06-19

**Authors:** Sedra Arnous, Rama Mdaghmesh, Alaa Rajab, Muaweia Barakat, Mohammad Al-Karaki, Sidra M Alali, Louei Nahas

**Affiliations:** 1 Faculty of Medicine, Department of Medicine and Surgery, Syrian Private University, Damascus, SYR; 2 Faculty of Medicine, Department of Medicine and Surgery, Division of Otorhinolaryngology, Syrian Private University, Damascus, SYR

**Keywords:** artificial intelligence, audiograms, chatgpt, diagnostic accuracy, gemini, hearing loss

## Abstract

Introduction: The utilization of large language models (LLMs) in healthcare shows promising potential for addressing the global shortage of audiologists. However, their effectiveness in understanding and interpreting complex, live, real-world structured audiological data requires further investigation, as previous studies have primarily focused on theoretical situations and scenarios. This study aimed to assess and compare the diagnostic inference abilities of ChatGPT (OpenAI, San Francisco, USA) and Gemini (Google, Mountain View, USA) in differentiating clinically confirmed diagnoses based on numerical pure-tone audiogram data.

Methods: This prospective comparative study was conducted at Damascus Hospital (Al-Mujtahid Hospital), Damascus, Syria. Pure-tone audiograms were collected from 309 patients aged between six and 90 years in the audiology department. Audiograms were converted into a numerical format and independently analyzed by ChatGPT-4o and Gemini 1.5 Pro. The outputs were then compared with the reference standard, namely, expert clinical diagnosis, to evaluate accuracy, specificity, sensitivity, and Cohen’s kappa for both models.

Results: Hearing loss was identified in 274 of 309 patients (88.7%). ChatGPT demonstrated 60.8% accuracy, with a weighted sensitivity of 68.9%, a weighted specificity of 94.5%, and a Cohen’s kappa of 0.578. Gemini achieved a significantly higher accuracy of 86.1%, with a weighted sensitivity of 87.7%, a weighted specificity of 97.5%, and a Cohen’s kappa of 0.848. Both models demonstrated high sensitivity and specificity for certain conditions, such as otosclerosis and presbycusis. The most common diagnosis was otosclerosis (27%), followed by presbycusis (16%).

Conclusion: The findings suggest that Gemini 1.5 Pro demonstrated stronger performance, achieving an accuracy of 86.1%, whereas ChatGPT-4o achieved 60.8% in interpreting real-world pure-tone audiograms. Both models showed high agreement in common audiometric patterns. These models should not be relied upon independently but may serve as supportive tools under clinician supervision to reduce diagnostic errors.

## Introduction

Hearing sensitivity assessment typically relies on pure-tone audiometry [1]. Results are presented as audiograms, showing hearing thresholds across different sound frequencies [1]. It helps diagnose the type and severity of hearing loss by evaluating and comparing air and bone conduction thresholds and noting the presence or absence of an air-bone gap (ABG), which assists physicians in distinguishing between conductive, sensorineural, and mixed hearing loss to reach an accurate diagnosis and management [2,3].

Hearing loss is classified into five degrees of severity: mild, moderate, moderately severe, severe, or profound [4]. Over 1.5 billion people now live with some degree of hearing loss [4].

Artificial intelligence (AI) is now considered a diagnostic aid for healthcare professionals, assisting rather than replacing human clinical decision-making, specifically by improving diagnostic precision [5]. 

Large language model (LLM)-based virtual assistants have been explored for health-related information delivery and clinical support tasks, exhibiting performance variation depending on the provided clinical task [6]. These models assist in diagnosis and tend to demonstrate more accurate results when the clinical scenarios are standardized [7]. A new machine learning approach was developed to automatically classify audiogram patterns [8]. This model demonstrated higher classification accuracy than traditional techniques [8]. Despite these developments, such tools remain rarely integrated into routine clinical practice.

Computational audiology and AI are rapidly evolving, yet their use in audiological practice is still limited [9,10]. Printed audiograms explicitly indicate hearing-level categories so healthcare specialists can interpret these charts using their experience. AI systems often face challenges in recognizing visual patterns. However, wider real-world testing and future trials are required before routine clinical use becomes possible [9].

Audiograms use symbols to distinguish between the right and left ears, as well as air and bone conduction responses [11]. The absence of explicit labeling could lead to misinterpretations, which may increase the risk of diagnostic errors [9]. Because of this, the conversion of graphical data into textual formats assists AI in interpreting audiometric information correctly [11].

Given the increasing use of AI in medical evaluation and the limited research on its performance in audiology, further study is needed [7]. This study evaluated the performance of ChatGPT (OpenAI, San Francisco, CA, USA) and Gemini (Google, Mountain View, CA, USA) [7,12] in interpreting pure-tone audiometry results. A total of 309 cases were converted into numerical format and analyzed. The AI diagnostic results were then evaluated against expert-confirmed diagnoses established after routine clinical evaluation.

This study aimed to evaluate and compare the approximate diagnostic accuracy of ChatGPT and Gemini, identify their respective limitations, and determine the clinical value of LLMs in audiologic interpretation [12]. Secondarily, model performance was analyzed across various age groups, sexes, and diagnostic categories. In the present study, it was hypothesized that when provided with standardized numerical pure-tone audiometric data, age, and sex, ChatGPT-4o and Gemini 1.5 Pro would differ in their ability to approximate expert-confirmed diagnoses, while functioning as audiometric pattern-recognition tools rather than independent systems for definitive clinical diagnosis. Accordingly, the present study should be interpreted within a digital decision-support framework, in which structured numerical audiometric data are processed by AI models to approximate diagnostic patterns, while final clinical interpretation remains dependent on human assessment.

## Materials and methods

Study design 

This prospective cross-sectional comparative study was conducted to evaluate the performance of LLMs (ChatGPT and Gemini) in interpreting structured pure-tone audiometric data and comparing their ability to approximate expert-confirmed diagnoses.

Study setting

Data were collected from the audiology department of Damascus Hospital (Al-Mujtahid Hospital), Damascus, Syria, between December 2025 and February 2026. 

The researchers attended the clinic daily and collected audiograms from patients during routine clinical evaluation. No personally identifiable information was collected; only age, sex, and audiometric data were recorded.

The collected data were then entered into ChatGPT-4o and Gemini 1.5 Pro to obtain their respective diagnoses. These outputs were subsequently evaluated against the expert-confirmed diagnoses provided by an expert clinician as the reference standard, in order to compare which model more closely approximated the confirmed diagnosis for each case.

Sample size and sampling method

A total of 309 audiograms were collected, with each audiogram including measurements for both the right and left ears. The sample used in the present study consisted of 145 females and 164 males, with ages ranging from six to 90 years.

Data were collected using a convenience sampling method with no exclusion criteria, whereby audiograms were obtained from all patients undergoing pure-tone audiometry during the study period.

Data collection procedure

Audiograms were collected daily from the audiology department at Damascus Hospital (Al-Mujtahid Hospital). A standardized prompt was used for all cases. Hearing thresholds were measured at the following frequencies: 125, 250, 500, 1000, 2000, 4000, 8000 Hz for air conduction, while bone conduction thresholds were measured at 250, 500, 1000, 2000, 4000 Hz. In addition, age and sex were recorded for each patient on the audiogram sheet because of their relevance in the differential diagnosis of certain conditions.

The audiograms were manually converted into numerical data, with hearing thresholds recorded for each frequency in both air and bone conduction for both ears. To ensure accuracy, a double data check was implemented, and any inconsistencies were reviewed and resolved through direct verification against the original audiograms. Subsequently, the numerical data were entered into ChatGPT-4o and Gemini 1.5 Pro. Each model received the same set of questions for each audiogram, which included the following: Is there hearing loss in each ear individually? If yes, what is the type of hearing loss in each ear? What is the severity of hearing loss in each ear? Is there an ABG? What is the probable diagnosis?

Audiograms were entered sequentially into the models in batches of five cases per newly initiated chat session. Each case was submitted individually, and the model response was recorded before proceeding to the next case. To ensure independent interpretation of each audiogram and to minimize short-term contextual memory effects, each session was introduced with a specific instruction to evaluate each case as an independent clinical entity. No feedback regarding correctness was provided between cases; however, limited contextual carryover within each five-case session could not be fully excluded because prior entries remained visible within the same chat. To reduce this effect, a new chat session was initiated after every batch of five. No prior diagnoses were revisited within the session.

At the beginning of each session, the following standardized prompt was used: “I will provide audiograms in numerical format. Evaluate each case as an independent clinical entity, then answer the listed diagnostic questions.” Both models were accessed through their official mobile applications under default inference settings available during the study period. When a model generated more than one possible diagnosis for question five, an additional instruction was issued requesting only the single diagnosis considered most likely by the model. The final predicted diagnoses generated by both models were compared against the reference diagnoses established by an expert otolaryngologist with over 18 years of clinical experience. The expert-confirmed diagnosis was based on audiogram interpretation together with routine clinical evaluation (e.g., patient history, otoscopy, Weber and Rinne tests) and relevant investigations (e.g., tympanometry, speech audiometry, CT or MRI scans) performed during patient assessment. This expert-based evaluation served as the clinical reference standard. Because only structured audiometric variables, age, and sex were provided, the models were evaluated for their ability to infer the most likely diagnosis from audiometric pattern recognition rather than to establish a definitive etiologic diagnosis independently.

A blinding approach was implemented, whereby the LLMs were not provided with the expert-confirmed diagnoses, and the expert was blinded to the LLMs’ suggested diagnoses. All data, including hearing thresholds at each frequency, presence of hearing loss, degree of severity, air-bone gap for each ear, the possible diagnoses generated by both models, and the expert-confirmed diagnoses, were systematically recorded in a structured Excel spreadsheet for analysis. The specific prompt and instructions provided to the AI models are available in Appendix A.

AI model interpretation

In the present study, ChatGPT-40 (OpenAI) and Gemini 1.5 Pro (Google DeepMind) were accessed between December 2025 and February 2026 using premium subscription accounts through their official applications. Both models were used through publicly available application interfaces rather than API-based access. Therefore, user-controllable inference parameters, including temperature, top-p, random seed, and related sampling settings, were not available and could not be fixed or recorded by the investigators. Each case was entered once without regeneration of responses. No response editing was performed before recording outputs, and outputs were recorded without any modifications immediately after generation. Repeated submissions of the same case were not performed; therefore, intra-model output consistency across repeated runs could not be assessed. Because publicly accessible LLM interfaces undergo periodic backend updates that are not fully user-controlled, exact reproducibility of outputs may vary over time, despite identical prompts and input structure.

Because definitive diagnosis of certain conditions requires clinical examination or ancillary investigations, the present study evaluated whether the models could infer possible etiologies from characteristic audiometric patterns rather than independently establish definitive diagnoses. For example, cerumen impaction and tympanic membrane perforation require physical examination, whereas an acoustic neuroma requires clinical correlation and imaging such as MRI. Since otoscopic findings, tympanometry, speech audiometry, CT, MRI, and other clinical data were not provided to the models, the digital system could not directly detect or confirm these conditions; it could only generate a probable diagnosis based on the structured audiometric thresholds, age, and sex.

Reference standard

An expert otolaryngologist with over 18 years of experience established the expert-confirmed diagnosis for each case based on audiogram interpretation together with routine clinical evaluation (e.g., patient history, otoscopy, Weber and Rinne tests) and relevant investigations (e.g., tympanometry, speech audiometry, CT, or MRI scans), which were performed during patient assessment, while remaining blinded to the outputs of both models.

The outputs generated by the AI models were then evaluated using the expert’s diagnosis as the reference standard to determine which model more closely approximated the confirmed diagnosis across cases. 

Statistical analysis

Statistical analysis was performed on 309 complete cases. This study followed a comparative diagnostic accuracy framework focused on approximation of expert-confirmed diagnoses.

Categorical variables were summarized as frequencies and percentages, while continuous variables were described using mean ± standard deviation (SD) for approximately normally distributed data, and median with interquartile range (IQR) where appropriate.

Hearing loss and its severity were classified according to the World Health Organization (WHO) standards. The WHO defines hearing loss as a pure-tone average (PTA) greater than 25 dB HL in the better-hearing ear [13,14]. Patient-level hearing loss prevalence was defined as the presence of hearing loss in either ear. Bilateral hearing loss was defined as hearing loss in both ears, and unilateral hearing loss as hearing loss in one ear only. For severity analyses, the worse ear was used to classify the overall degree of hearing loss.

The AI-generated approximate diagnoses from ChatGPT and Gemini were each compared against the expert-confirmed diagnoses on a case-by-case basis. Head-to-head comparison of approximate diagnostic correctness between the two models was then performed using McNemar’s test. Diagnostic label harmonization was performed according to a predefined diagnostic-category dictionary before calculation of the grouped-category performance metrics. This process was based on clinical equivalence and synonymy rather than model performance. Minor orthographic and formatting variations, including capitalization, spacing, spelling differences, abbreviations, and singular/plural wording, were first standardized. Diagnostically equivalent or closely synonymous labels were then mapped to the same broader diagnostic category when they represented the same underlying clinical entity. For example, “normal hearing,” “normal audiogram," and "no hearing loss” were grouped as normal audiogram; “age-related hearing loss” and "presbycusis" were grouped as presbycusis; "middle-ear effusion" and "otitis media with effusion" were grouped as otitis media with effusion; and "Eustachian tube dysfunction" and "ETD" were grouped as eustachian tube dysfunction. Exact-match analysis was retained as the stricter primary comparison, whereas grouped-category matching was used as a secondary, clinically oriented analysis to reduce the impact of wording variability in free-text Al-generated diagnoses.

For each model, diagnostic approximation accuracy was calculated as the proportion of correctly classified cases out of the total number of cases. Agreement beyond chance between each AI model and the expert-confirmed diagnosis was assessed using Cohen’s kappa coefficient.

Because the final diagnosis was a multiclass categorical outcome, diagnostic approximation sensitivity and specificity were evaluated using a one-vs-rest approach for each diagnostic category. For each class, the diagnosis of interest was treated as the positive class, and all remaining diagnoses were treated as the negative class. Class-specific sensitivity, specificity, positive predictive value (PPV), and negative predictive value (NPV) were calculated. To ensure statistical stability despite the small sample sizes in certain categories, cases with low frequencies were aggregated into an ‘Other Rare Diagnoses’ group. Furthermore, macro averages were computed to treat all diagnostic classes equally, providing an assessment of model performance on rare conditions, whereas weighted averages were adjusted for the prevalence of each condition to reflect overall clinical utility in the study-specific sample. Differences in diagnostic approximation between the two models were assessed using McNemar’s test for paired binary outcomes to assess differences in accuracy. A two-sided p-value <0.05 was considered statistically significant. Because several diagnostic categories had limited sample sizes, 95% confidence intervals for proportions were calculated using the Wilson score method. All analyses were performed using IBM SPSS Statistics software for Mac, version 27 (IBM Corp., Armonk, NY, USA).

Ethics

Ethical approval was obtained from the Research Ethics Committee at the Syrian Private University (Approval No. MD-24112025-117) on 24 November 2025.

Data were collected from the audiology department at Damascus Hospital. Strict measures were implemented to safeguard patient confidentiality. No personally identifiable information was collected; only age, sex, and audiometric data were recorded. All procedures were conducted in accordance with established ethical guidelines. The data were stored in a password-protected Microsoft Excel spreadsheet (Microsoft Corp., Redmond, WA, USA), with access restricted solely to the research team. The data were utilized exclusively for research purposes.

## Results

Study population and baseline characteristics

A total of 309 audiograms were included in the analysis. The mean age was 45.1 years (SD 17.3), with an age range of six to 90 years. There were 164 males (53.1%) and 145 females (46.9%).

Hearing loss was identified in 274 patients (88.7%), while 35 (11.3%) had no hearing loss in either ear. Hearing loss was bilateral in 232 patients (75.1%) and unilateral in 42 (13.6%). At the ear level, hearing loss was present in 252 right ears (81.6%) and 254 left ears (82.2%).

Severity was assessed at the patient level according to the worse-hearing ear. One hundred and ten patients (35.6%) had severe/profound hearing loss, 110 (35.6%) had moderate/moderately severe hearing loss, 57 (18.4%) had mild hearing loss, and 32 (10.4%) had no hearing loss.

The most common ear-specific type of hearing loss was mixed hearing loss, affecting 158 right ears (51.1%) and 148 left ears (47.9%). An air-bone gap was present in 224 right ears (72.5%) and 212 left ears (68.6%).

**Figure 1:**
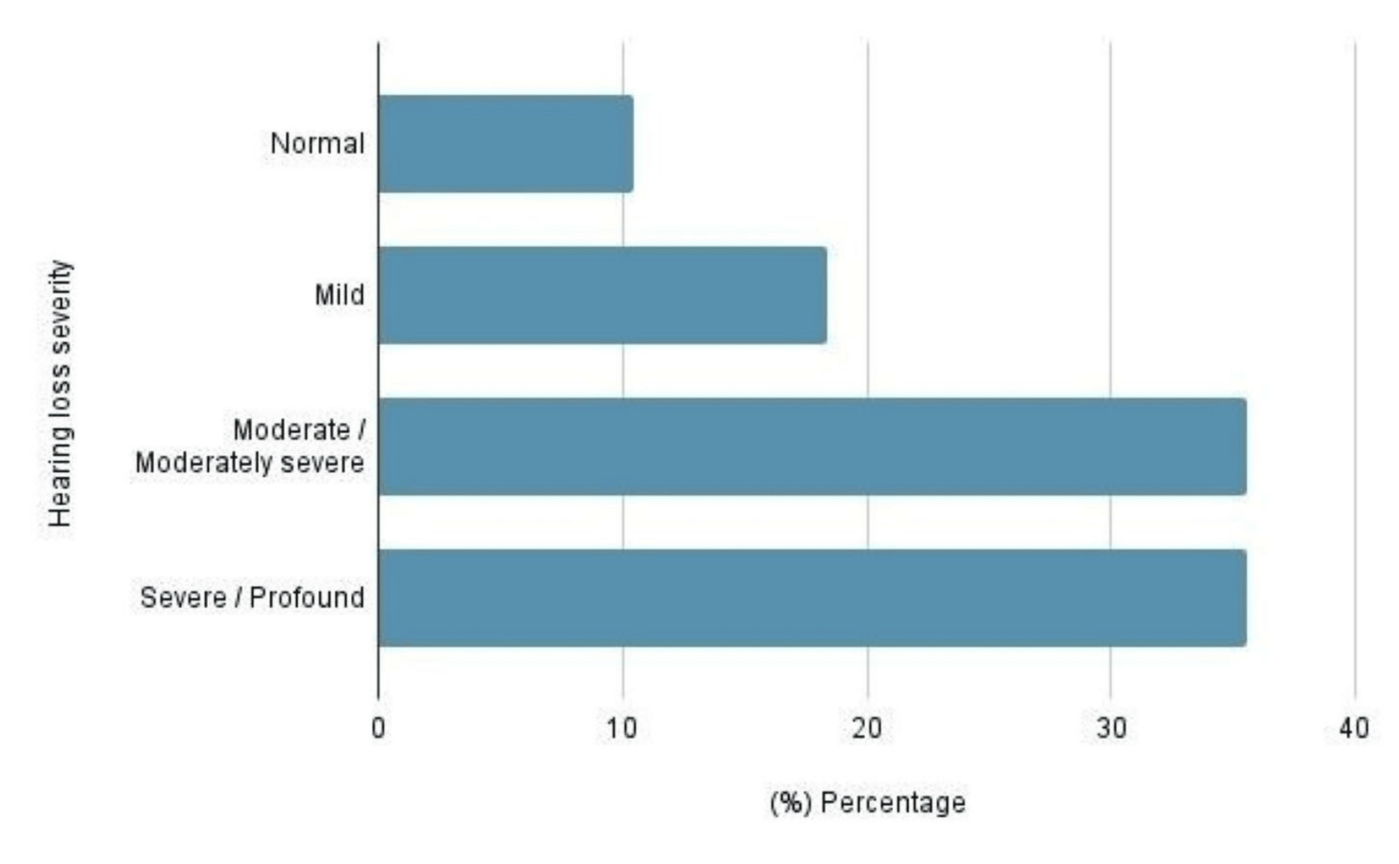
Distribution of hearing loss severity. The majority of cases were classified as severe to profound and moderate to moderately severe (35.6%). Patient-level hearing loss was defined as hearing loss in either ear. Worst-ear severity was based on the more severely affected ear.

Baseline demographic and hearing characteristics are shown in Table 1 and Figure 1, while ear-specific audiometric findings are summarized in Table 2. 

**Table 1:** Ear-specific audiometric characteristics.

Characteristics	Right ear, n (%)	Left ear, n (%)
Hearing loss type		
Normal	54 (17.5%)	52 (16.8%)
Conductive hearing loss	50 (16.2%)	44 (14.2%)
Sensorineural hearing loss	47 (15.2%)	65 (21.0%)
Mixed hearing loss	158 (51.1%)	148 (47.9%)
Severity category		
Normal	55 (17.8%)	52 (16.8%)
Mild	61 (19.7%)	60 (19.4%)
Moderate/Moderately severe	123 (39.8%)	117 (37.9%)
Severe/Profound	70 (22.7%)	80 (25.9%)
Air-bone gap		
Present	224 (72.5%)	212 (68.6%)
Absent	85 (27.5%)	97 (31.4%)

**Table 2:** Baseline characteristics of the study population (n = 309). *Percentages are calculated using 309 patients as the denominator for each ear.

Characteristic	Value
Total number of included patients	309
Mean age, years (SD)	45.1 (17.3)
Median age, years (IQR)	45 (33-59)
Age range, years	6-90
Male sex, n (%)	164 (53.1%)
Female sex, n (%)	145 (46.9%)
Hearing loss in at least one ear, n (%)	274 (88.7%)
No hearing loss in either ear, n (%)	35 (11.3%)
Bilateral hearing loss, n (%)	232 (75.1%)
Unilateral hearing loss, n (%)	42 (13.6%)
Right ear hearing loss, n (%)	252 (81.6%)
Left ear hearing loss, n (%)	254 (82.2%)

Distribution of hearing loss by sex and age

**Figure 2:**
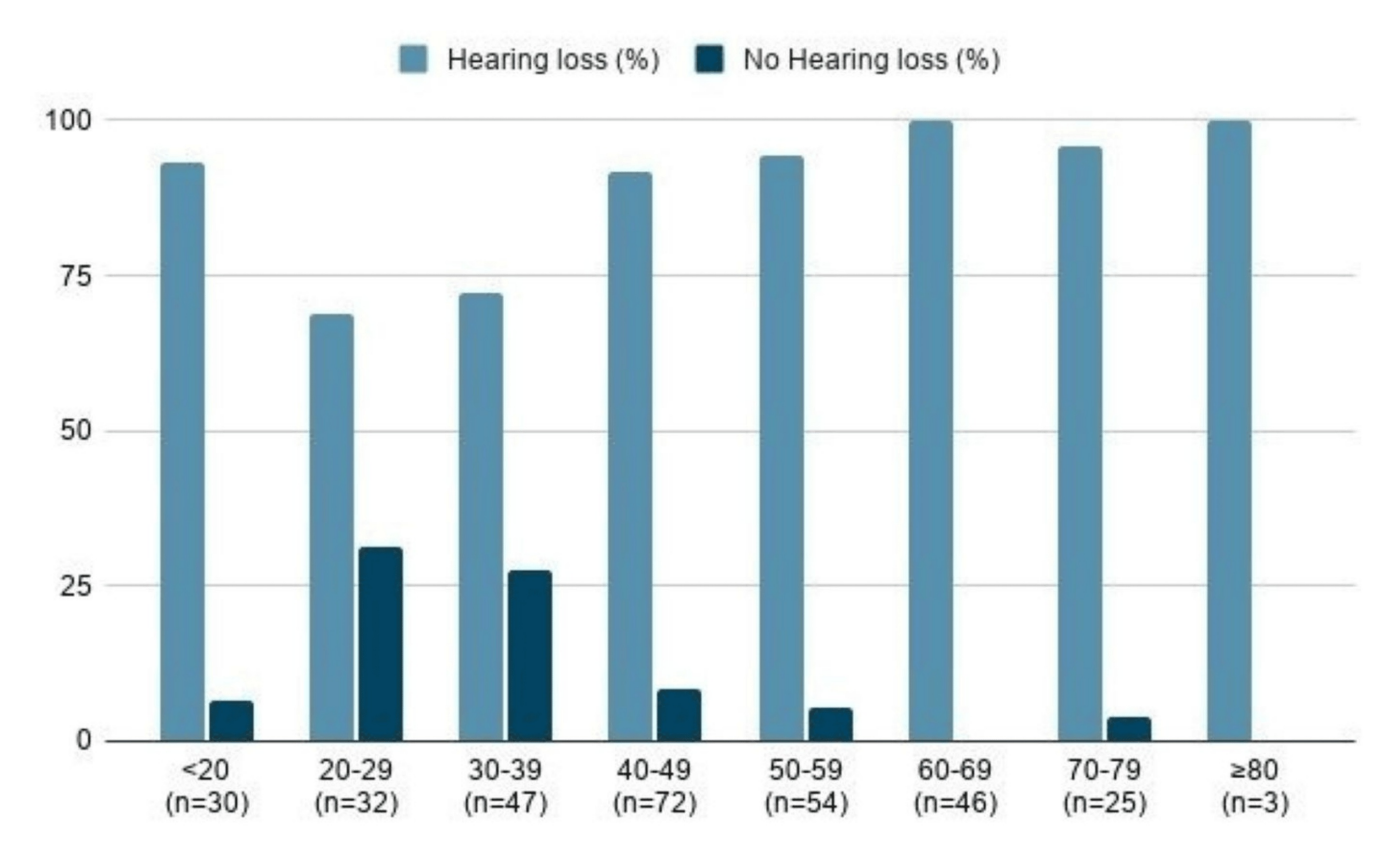
Distribution of hearing loss by age group. The light blue column shows patients with hearing loss, and the dark blue column shows patients without hearing loss. (n) : total number of patients in each age group.

The prevalence of hearing loss in at least one ear was high in both sexes, affecting 126 of 145 females (86.9%) and 148 of 164 males (90.2%), as shown in Table 3. Although the prevalence was slightly higher in males, this difference was not statistically significant (χ² = 0.86, p = 0.354). Patients with hearing loss were older on average than those without hearing loss (mean age 46.5 vs. 34.4 years, respectively), as shown in Table 4 and Figure 2.

**Table 3:** Distribution of hearing loss by sex.

Sex	Total (n)	Hearing loss n (%)	No hearing loss n (%)
Male	164	148 (90%)	16 (9.8%)
Female	145	126 (86%)	19 (13.1%)

**Table 4:** Distribution of hearing loss by age group.

Age group (years)	Total (n)	Hearing loss (%)	No hearing loss (%)
<20	30	93.3%	6.7%
20-29	32	68.8%	(31.2%)
30-39	47	72.3%	27.7%
40-49	72	91.7%	8.3%
50-59	54	94.4%	5.6%
60-69	46	100%	0%
70-79	25	96.0%	4.0%
≥80	3	100%	0%

Expert-confirmed diagnoses

Using grouped diagnostic categories, the most frequent expert-confirmed diagnosis was otosclerosis, identified in 86 cases (27.8%), followed by presbycusis (50 cases, 16.2%), normal audiogram (38 cases, 12.3%), and chronic otitis media (35 cases, 11.3%). Other less frequent diagnoses included noise-induced hearing loss (16 cases, 5.2%), other hearing loss diagnoses (12 cases, 3.9%), otitis media with effusion (10 cases, 3.2%), cerumen impaction (10 cases, 3.2%), eustachian tube dysfunction (nine cases, 2.9%), otitis (seven cases, 2.3%), and combined presbycusis and otosclerosis (seven cases, 2.3%). In addition to the previous categories, several rarer diagnostic categories were represented by only a small number of cases (29 cases, 9.4%) (Table 5).

**Table 5:** The distribution of expert-confirmed diagnoses. *Other rare diagnoses included low-frequency categories such as congenital or genetic hearing loss, trauma, ossicular fixation, ossicular discontinuity, tympanosclerosis, chronic suppurative otitis media, sudden sensorineural hearing loss, tympanic membrane perforation, Ménière’s disease, labyrinthitis, and acoustic neuroma.

Expert-confirmed diagnosis	n (%)
Otosclerosis	86 (27.8%)
Presbycusis	50 (16.2%)
Normal audiogram	38 (12.3%)
Chronic otitis media	35 (11.3%)
Noise induced hearing loss	16 (5.2%)
Other hearing loss diagnosis	12 (3.9%)
Otitis media with effusion	10 (3.2%)
Cerumen impaction	10 (3.2%)
Eustachian tube dysfunction	9 (2.9%)
Otitis	7 (2.3%)
Presbycusis + Otosclerosis	7 (2.3%)
Other rare diagnosis	29 (9.4%)

Diagnostic agreement between AI models and the expert-confirmed diagnosis

The summary diagnosis generated by each AI model was compared against the expert-confirmed diagnosis on a case-by-case basis. Using an exact-match approach after standardization of diagnostic labels, ChatGPT generated approximate diagnostic labels matching the expert-confirmed diagnosis in 188 cases (60.8%, 95% CI: 55.3-66.1%), whereas Gemini produced outputs matching the expert-confirmed diagnosis in 266 cases (86.1%, 95% CI: 81.8-89.5%).

Agreement beyond chance, assessed using Cohen’s kappa, was κ = 0.578 (95% CI: 0.522-0.640%) for ChatGPT and κ = 0.848 (95% CI: 0.805-0.890%) for Gemini. Because free-text diagnoses may vary in wording despite representing the same underlying category, an additional grouped category analysis was performed. Under this broader diagnostic classification, ChatGPT matched the final diagnosis category in 213 cases (68.9%, 95% CI: 63.6-73.8%), while Gemini matched in 271 cases (87.7%, 95% CI: 83.6-90.9%). Corresponding kappa values were κ = 0.634 for ChatGPT and κ = 0.855 for Gemini. Direct comparison of the two models showed that Gemini demonstrated significantly higher approximate diagnostic matching than ChatGPT relative to the final confirmed diagnosis (McNemar test p < 0.001). These metrics are summarized in Table 6.

**Table 6:** Audiometric hearing loss pattern interpretation and approximate diagnostic performance of ChatGPT and Gemini against the expert-confirmed diagnosis.

Metric	ChatGPT	Gemini
Exact-match correct cases, n/N	188/309	266/309
Exact-match accuracy, % (95% CI)	60.8 (55.3–66.1)	86.1 (81.8–89.5)
Cohen’s kappa for exact diagnosis (95% CI)	0.578 (0.522–0.640)	0.848 (0.805–0.890)
Category-match correct cases, n/N	213/309	271/309
Category-match accuracy, % (95% CI)	68.9 (63.6–73.8)	87.7 (83.6–90.9)
Cohen’s kappa for grouped diagnosis category	0.634	0.855
Weighted sensitivity, %	68.9	87.7
Weighted specificity, %	94.5	97.5
Macro sensitivity, %	48.3	78.7
Macro specificity, %	98.4	99.4

Sensitivity and specificity of AI approximate diagnostic classification

Diagnostic agreement across categories is summarized in Table 7.

**Table 7:** Class-specific approximate diagnostic performance for common final diagnoses. PPV: positive predictive value; NPV: negative predictive value.

Diagnosis category	Model	Sensitivity % (95% CI)	Specificity % (95% CI)	PPV % (95% CI)	NPV % (95% CI)
Otosclerosis	ChatGPT	79.1 (68.9–86.6)	87.0 (81.9–90.8)	72.3 (62.1–80.6)	90.8 (86.0–94.1)
	Gemini	97.7 (91.9–99.4)	90.1 (85.4–93.4)	80.0 (71.2–86.7)	98.8 (95.8–99.7)
Presbycusis	ChatGPT	86.0 (73.8–93.1)	93.8 (90.1–96.2)	72.9 (60.3–82.7)	97.2 (94.4–98.6)
	Gemini	98.0 (89.5–99.6)	99.2 (97.1–99.8)	96.1 (86.8–98.9)	99.6 (97.8–99.9)
Normal audiogram	ChatGPT	84.2 (69.6–92.6)	99.3 (97.3–99.8)	94.1 (80.9–98.4)	97.8 (95.3–99.0)
	Gemini	94.7 (82.7–98.5)	99.6 (97.9–99.9)	97.3 (86.2–99.5)	99.3 (97.4–99.8)
Chronic otitis media	ChatGPT	77.1 (61.0–87.9)	96.0 (92.9–97.8)	69.2 (53.6–81.5)	97.2 (94.4–98.6)
	Gemini	82.9 (67.3–91.9)	99.3 (97.4–99.8)	93.5 (79.3–98.2)	97.8 (95.3–99.0)
Noise-induced hearing loss	ChatGPT	81.2 (57.0–93.4)	97.6 (94.9–98.9)	68.4 (46.0–84.6)	98.9 (96.9–99.6)
	Gemini	68.8 (44.4–85.8)	99.7 (98.1–99.9)	91.7 (64.6–98.5)	98.3 (96.1–99.2)
Otitis media with effusion	ChatGPT	20.0 (5.7–51.0)	99.7 (98.1–99.9)	66.7 (20.8–93.9)	97.4 (95.0–98.7)
	Gemini	100.0 (72.2–100.0)	99.3 (97.4–99.8)	83.3 (55.2–95.3)	100.0 (98.7–100.0)
Cerumen impaction	ChatGPT	30.0 (10.8–60.3)	99.7 (98.1–99.9)	75.0 (30.1–95.4)	97.7 (95.4–98.9)
	Gemini	90.0 (59.6–98.2)	100.0 (98.7–100.0)	100.0 (70.1–100.0)	99.7 (98.1–99.9)
Eustachian tube dysfunction	ChatGPT	55.6 (26.7–81.1)	99.7 (98.1–99.9)	83.3 (43.6–97.0)	98.7 (96.8–99.5)
	Gemini	88.9 (56.5–98.0)	99.7 (98.1–99.9)	88.9 (56.5–98.0)	99.7 (98.1–99.9)

As the diagnostic outcome was multiclass rather than binary, agreement performance was also assessed using a one-vs-rest approach for each diagnosis category, with summary metrics reported as weighted and macro-averaged values.

For ChatGPT, the weighted sensitivity was 68.9%, and the weighted specificity was 94.5%. The macro sensitivity was 48.3%, and the macro specificity was 98.4%. For Gemini, the weighted sensitivity was 87.7%, and the weighted specificity was 97.5%. The macro sensitivity was 78.7%, and the macro specificity was 99.4%.

For the most frequent diagnosis, otosclerosis, ChatGPT achieved a sensitivity of 79.1% (95% CI: 68.9-86.6%) and specificity of 87.0% (95% CI: 81.9-90.8%), whereas Gemini achieved a sensitivity of 97.7% (95% CI: 91.9-99.4%) and specificity of 90.1% (95% CI: 85.4-93.4%). For presbycusis, ChatGPT achieved sensitivity and specificity of 86.0% (95% CI: 73.8-93.1%) and 93.8% (95% CI: 90.1-96.2%), respectively, compared with 98.0% (95% CI: 89.5-99.6%) and 99.2% (95% CI: 97.1-99.8%) for Gemini. Gemini also showed higher performance across several other common categories, including cerumen impaction, eustachian tube dysfunction, and otitis media with effusion, although performance varied in rarer diagnostic classes. To improve the transparency of statistical estimation, particularly for categories with limited sample size, exact confidence intervals for all class-specific diagnostic metrics and full multiclass confusion matrices are provided in Appendix B.

Categorical metrics and classification accuracy

Detailed analysis among 19 clinical categories demonstrated that Gemini 1.5 Pro and ChatGPT-4o maintained high specificity (>95%) in most classes, while a notable disparity was observed in sensitivity levels between both models. Gemini demonstrated stable results across different categories, while ChatGPT’s performance dropped in specific cases, such as otitis media with effusion, failing to identify the majority of true-positive cases and resulting in a high false-negative rate. The complete diagnostic confusion matrices for both models are provided in Appendix B. 

## Discussion

This study evaluated the accuracy of LLMs (ChatGPT and Gemini) in interpreting and analyzing numerical audiometric data and compared their approximate diagnoses with final confirmed diagnoses, which were given by an expert. The study included 309 patients with a mean age of 45.1 years (SD 17.3): 164 males and 145 females. The majority of patients had severe to profound or moderate to moderately severe hearing loss, as shown in Figure 1. Most cases had mixed hearing loss affecting 158 right ears and 148 left ears, followed by sensorineural hearing loss affecting 47 right and 65 left ears, which is consistent with the findings of Kassjański et al. [15]. The demographic distribution of the present study sample allowed the evaluation of LLMs across a range of age- and sex-associated conditions.

Generally, Gemini showed a significantly higher exact-match accuracy of 86.1% (95% CI: 81.8-89.5%) compared with ChatGPT's 60.8% (95% CI: 55.3-66.1%). In addition, category-match accuracy was used as a less strict evaluation reflecting the ability of each model to identify the general diagnostic category. Gemini outperformed ChatGPT even though ChatGPT showed an improvement in performance of 87.7% (95% CI: 83.6-90.9%) vs. 68.9% (95% CI: 63.6-73.8%).

Weighted and macro sensitivity were both higher for Gemini (87.7% and 78.7%) compared to ChatGPT (68.9% and 48.3%), which confirms that Gemini outperformed ChatGPT in identifying both rare and common cases. In addition, higher weighted and macro specificity were observed for Gemini (97.5% and 99.4%) compared to ChatGPT (94.5% and 98.4%). However, Gemini slightly outperformed ChatGPT in terms of specificity, which suggests that both models were capable of identifying non-target diagnoses accurately.

The most common diagnosis in the study was otosclerosis (86 cases, 27%), followed by presbycusis (50 cases, 16.2%). Both models demonstrated high agreement and performance in these diagnostic categories, which can be explained by the presence of recognized audiometrical patterns, such as the downward slope at high frequencies in presbycusis [1] and the Carhart notch in otosclerosis (an artificial dip in bone conduction threshold most prominent at 2000 Hz) [1]. Class-specific sensitivity, specificity, PPV, and NPV values for both models are presented in Table 7.

Higher ChatGPT performance was observed in certain conditions such as noise-induced hearing loss; the sensitivity reached 81.2% (95% CI: 57.0-93.4%) and specificity reached 97.6% (95% CI: 94.9-98.9%) compared to Gemini, which achieved 68.8% (95% CI: 44.4-85.8%) sensitivity and 99.7% (95% CI: 98.1-99.9%) specificity. The performance of both models reached high levels in certain conditions, such as eustachian tube dysfunction and otitis media, where both models demonstrated consistently high specificity of 99.7% (95% CI: 98.1-99.9%). Although differences were observed in sensitivity, which was higher for Gemini (88.9%, 95% CI: 56.5-98.0). 

Overall, specificity was largely similar between both models across certain conditions, which indicates that both models have a similar ability in most conditions to identify non-target diagnoses. In contrast, sensitivity showed a more pronounced difference favoring Gemini, suggesting superior ability to identify target diagnostic categories within the one-vs-rest classification framework. The observed difference between the models may reflect the previously reported strength of Gemini models in structured medical reasoning and complex clinical inference tasks [16], although the precise mechanism underlying this performance difference in audiometric interpretation cannot be determined from the present study.

Previous studies have reported findings that differ from the present study’s result by showing that ChatGPT demonstrated higher accuracy in certain medical conditions [17]. This inconsistency may be attributed to differences in prompt design or data input, as the previous study relied on using image-based data such as chest radiology and ultrasound [17]. However, the same study demonstrated that Gemini outperformed ChatGPT in specific medical conditions, including renal disorders, low potassium, and high phosphorus scenarios. This suggests that both models have their own strengths in particular medical domains [17].

Despite the promising potential and the continuous improvements in AI, it remains an insufficient tool for standalone diagnosis, especially when only one component of diagnosis, such as pure-tone audiometry, is used, while clinical decision-making still relies on experts. AI should be considered a supportive tool rather than a replacement for physician expertise. 

Therefore, combining the strength of both models and the expertise of specialized clinicians may contribute to the future development of more accurate and reliable diagnostic systems to help both patients and specialists.

There are several study limitations to consider. Although efforts were made to maintain consistency across all cases, prompt phrasing remains a possible source of variability in LLM performance. Moreover, during January, an interruption of two weeks occurred in data collection procedures due to the temporary closure of the audiology department for renovation and structural repair. This closure extended the study duration and limited the final sample size. Some diagnostic categories had a limited number of patients in the dataset, which may result in unstable estimates of sensitivity and specificity. In such cases, high performance metrics (e.g., 100% sensitivity) should be interpreted with caution. Further studies with sufficient sample sizes are needed and recommended to elicit reliable estimates and results.

A noteworthy limitation is that the LLMs received only structured audiometric thresholds together with age and sex, whereby clinically referenced diagnoses included additional examination findings and investigations as available. Consequently, the current results are better interpreted as partial agreement with clinically confirmed diagnoses under constrained input conditions rather than evidence of individual diagnostic equivalence.

Moreover, pure-tone audiometry itself is not a precisely specific diagnostic tool for certain conditions. Acoustic neuroma and cerumen impaction, for example, cannot be definitively diagnosed from pure-tone audiometry alone, because acoustic neuroma requires clinical correlation and imaging such as MRI, while cerumen impaction requires physical examination. Therefore, the AI models’ performance should be understood as audiometric pattern recognition within a restricted data context rather than direct detection or confirmation of these conditions. Finally, structured numerical data were used as the input format instead of raw audiometric images.

An additional limitation relates to potential residual contextual carryover within the five-case chat sessions. Although each session included an instruction to evaluate each audiogram as an independent clinical entity, and a new chat session was initiated after every five cases, previous prompts and model responses remained visible within the same active conversation. Therefore, complete independence between cases could not be guaranteed. This could potentially influence diagnostic performance and reproducibility by priming the models toward recently generated diagnostic patterns, terminology, or reasoning pathways. Such carryover could theoretically have improved apparent consistency when consecutive cases shared similar audiometric features or, conversely, contributed to misclassification if prior outputs biased subsequent diagnostic label selection. Because no expert-confirmed diagnoses, correctness feedback, or corrective information were provided between cases, any potential carryover was limited to the models' own previous outputs rather than external diagnostic feedback. Although the same batching procedure was applied to both models, model-specific susceptibility to contextual carryover cannot be excluded.

Finally, the clinical reference standard was based on the diagnosis of a single senior otolaryngologist with extensive experience. An independent secondary review was considered during study planning; however, formal second-rater adjudication was not performed because the reference diagnoses were obtained from routine clinical evaluations conducted by a single senior clinician within their existing clinical workflow, which did not provide a structured opportunity for independent re-assessment. Consequently, inter-rater agreement for the reference standard could not be calculated. To limit potential reference-standard bias, the expert-confirmed diagnoses were not only based on pure-tone audiometry but also on routine clinical assessment and, where required, additional investigations. Moreover, the expert remained blinded to the AI-generated outputs throughout the evaluation. Nevertheless, potential variability in expert interpretation cannot be fully excluded.

Future studies should incorporate independent assessment by multiple otolaryngologists or audiology specialists, with consensus adjudication and inter-rater agreement analysis, to strengthen the reliability of the reference standard. Methodological reproducibility could be further improved by evaluating each case within a separate, independent session, randomizing case order, and employing stateless API-based calls to preserve case independence.

To reduce these risks, it is important to maintain human expert oversight when using these systems. Future research should evaluate these models in multiple centers using larger and more diverse datasets and should extend beyond structured PTA thresholds alone. In particular, future studies should compare LLM-based interpretation with other digital audiology systems, including audiogram image-analysis tools, machine-learning audiogram classifiers, and multimodal decision-support systems that can integrate PTA results with otoscopy, tympanometry, speech audiometry, imaging findings, and relevant clinical history. Such comparisons would help clarify whether LLMs are most useful as standalone text-based pattern-recognition tools, as components of broader digital audiology platforms, or as adjuncts within clinician-led medical interpretation workflows.

Furthermore, the global shortage of ear and hearing specialists limits timely access to care for people, especially in low-resource areas [18]. Advanced AI tools may help support initial audiometric interpretation in settings where specialist access is limited; however, their future clinical value will depend on safe integration with human assessment, validation against established digital systems, and clear distinction between preliminary audiometric interpretation and definitive medical diagnosis.

## Conclusions

Although pure-tone audiometry remains the cornerstone for assessing hearing loss, integrating AI shows promising potential for improving the interpretation and accessibility of audiometric data. Importantly, the variable performance of the models in less common diagnostic categories indicates the need for cautious and clinically supervised implementation. The comparative findings indicate that model performance may vary when LLMs are used to approximate clinically confirmed diagnoses from structured pure-tone audiometric data. Because the models received only structured audiometric thresholds, age, and sex, the findings should be interpreted as evidence of diagnostic approximation based on audiometric pattern recognition rather than evidence of independent, definitive clinical diagnosis. According to the present findings, LLMls, particularly Gemini, may be selectively used as adjunctive tools for structured audiometric interpretation under specialist supervision and oversight rather than as independent diagnostic systems. Nevertheless, key challenges related to data representation, input standardization, and accurate recognition of overlapping audiometric patterns remain unresolved. This study, therefore, contributes to the emerging evidence base by systematically evaluating two contemporary LLMs and their potential role in supporting patient-centered audiological care using real-world clinical audiograms. AI should be regarded as a complementary aid rather than a substitute for independent clinical judgment. Future research should focus on validating these models across larger and more diverse populations, multicenter clinical settings, and standardized input frameworks to facilitate safe and effective integration into routine audiology practice.

## Appendices

Appendix A

The specific prompts and instructions provided to the AI models are available at the following DOI: https://doi.org/10.6084/m9.figshare.32243169: 

Appendix B

The complete diagnostic confusion matrices for both models are provided in the following DOI: https://doi.org/10.6084/m9.figshare.32243169: 
